# An invasive infection caused by the thermophilic mold *Talaromyces thermophilus*

**DOI:** 10.1007/s15010-021-01648-z

**Published:** 2021-06-30

**Authors:** Karl Dichtl, Özlem Koc, Johannes Forster, Christina Scharf, Sebastian Suerbaum, Joachim Andrassy, Johannes Wagener, Ines Schroeder

**Affiliations:** 1 Max von Pettenkofer-Institut für Hygiene und Medizinische Mikrobiologie, Medizinische Fakultät, LMU München, Munich, Germany; 2 Institut für Hygiene und Mikrobiologie, Julius-Maximilians-Universität Würzburg, Würzburg, Germany; 3 Department of Anesthesiology, LMU Hospital, Munich, Germany; 4 Department of General, Visceral, and Transplant Surgery, LMU Hospital, Munich, Germany; 5 Department of Clinical Microbiology, School of Medicine, Trinity College Dublin, The University of Dublin, St James’s Hospital Campus, Dublin, Ireland; 6 Nationales Referenzzentrum für Invasive Pilzinfektionen (NRZMyk), Jena, Germany

**Keywords:** *Talaromyces*, Invasive fungal infection, Thermophile, Antigen testing, Serology

## Abstract

**Background:**

Increasing incidence of invasive infections caused by rare fungi was observed over the recent years.

**Case:**

Here, we describe the first reported case of an infection caused by the thermophilic mold *Talaromyces thermophilus*. Cultivation and, hence, identification of this fastidious organism is challenging since standard incubation conditions are not sufficient. Retrospective analysis of patient samples and in vitro experiments demonstrated that testing for fungal antigens, i.e., the cell wall components galactomannan and β-1,3-d-glucan, is a promising tool.

## Introduction

Invasive mold infections (IMI) are a major threat for immunosuppressed patients with mortality rates of up to 95% [1, 2]. Particularly, individuals undergoing myeloablative chemotherapy and hematopoietic stem cell transplantation (HSCT) are considered a high-risk population [1]. However, the incidence in non-canonical risk patients was also found to be rising over the recent years, e.g., in individuals in need of intensive care treatment, with a history of solid organ transplantation, influenza, or less severe regimens of immunosuppression [1].

Notably, this diversification of risk factors comes along with a diversification of the pathogen spectrum: Besides the most common cause of IMI, i.e., members of the genus *Aspergillus*, a broad range of other well-known but also emerging molds was more and more frequently identified in invasive fungal disease [3–7]. While the diagnostic assays to detect invasive aspergillosis (IA) have been refined over the past decades, we still lack appropriate tools to meet the challenge of diagnosing these new pathogens [3–5, 7–9]. To date, specific serologic assays for non-*Aspergillus*-IMI are not commercially available [8]. Molecular methods for analysis of clinical specimens still suffer from a lack of standardization and are either not recommended by current guidelines or only with restrictions [3–5]. The prospects of success of cultivation highly depend on the nature of the fungus: the need for special handling in the preanalytical phase and specific growth conditions, e.g., prolonged incubation, complicate the diagnosis of IMI [4, 5, 7]. Particularly incubation at 37 °C, which is the standard condition in medical microbiology, is a major contributor to false-negative cultures since this temperature is too high to sustain growth of many molds [5, 7]. Contrarily, there are exceptional cases of infections due to thermophilic organisms that require high incubation temperatures.

To the best of our knowledge, we present the first case of an infection caused by the thermophilic mold *Talaromyces thermophilus*. We outline not only possibilities but also limitations for diagnosing this rare pathogen. Additional analyses and in vitro experiments elucidate the potential and pitfalls of antigen testing for this exceptional IMI.

## Case

At the age of 25 years, a male Caucasian was diagnosed with an angiomyxoma located in the head of the pancreas and the hepatic hilum. Due to the constantly growing tumor, several complications and life-threatening events occurred, e.g., portal vein occlusion, ascites, and several episodes of upper gastrointestinal bleeding. Since all conservative therapy approaches were exhausted after four years of therapy, the patient consented to a radical tumor resection and multivisceral transplantation including liver, pancreas, stomach, small intestines, and colon ascendens (day 0). The patient received a calcineurin inhibitor-based immunosuppression combined with prednisolone and mycophenolate. However, the postoperative course was complicated by numerous drawbacks like primary transplant failure of the liver and pancreas, thrombophilia with thrombosis of the inferior vena cava and the portal vein, anastomotic leakage, and multiple septic episodes. As a result, the patient underwent several procedures and operations. Pancreatectomy, colectomy, total gastrectomy, and distal esophagectomy were performed on day 53 with the aim to regain infection control. On day 61, the patient received a second liver transplant, which again failed to provide sufficient function in the further course.

From day 67 on, several serum samples were tested positive for *Aspergillus* antigen galactomannan (GM; cut-off index: 0.50). At this point, the patient was already receiving caspofungin for three weeks because of continuous intestinal leakage and a previous finding of *Candida* spp. in the stool. Furthermore, *Candida albicans* had been recovered from a superficial swab of the abdominal wound (day 37). Upon the report of GM antigenemia, *Aspergillus*-specific treatment with liposomal amphotericin B (3 mg / kg once daily) was added to the therapeutic regime (day 68).

However, the patient, who was intubated and ventilated, did not have signs of respiratory tract infection, and bronchoalveolar lavage did not yield growth of *Aspergillus* spp. and was tested negative for GM (cut-off index: 0.50). Since antigenemia remained the only sign of IA, we tried to identify a cause for GM false-positivity. The day before the first positive serology, the digestion enzyme mix Nortase® (Repha, Langenhagen, Germany) was administered via a feeding tube to compensate the patient’s pancreatic insufficiency (day 66). The encapsulated powder contains fungal enzymes synthesized by *Rhizopus oryzae* and by *Aspergillus oryzae*. Therefore, powder diluted in 20 mL of water was tested and yielded a strongly positive reaction in the GM ELISA (Platelia *Aspergillus* GM ELISA, Bio-Rad Laboratories, Hercules, CA, USA). Upon this result, amphotericin B was discontinued, as antigenemia was considered to be a result of Nortase® administration [10].

An unexpected turn of events occurred, when two intra-abdominal samples again proved the initial suspicion of IMI: On day 71, peritoneal fluid was found to contain 260 neutrophils/µl and to be strongly reactive (index: 4.86) in the GM ELISA, which was accompanied by a peak neutrophil leukocyte count of 30 G/L. Over the following days, leukocytes dramatically dropped to 0.9 G/L. After nine days of incubation (day 74), a mold was recovered from a swab of a suspicious alteration of the abdominal mesh, which had been inserted on day 47, during jejunostomy for a biliodigestive anastomosis. This mold was identified as *Talaromyces thermophilus* (synonyms: *T. dupontii* or *Thermomyces dupontii;* anamorph: *Penicillium dupontii*) via MALDI-TOF (Bruker Corporation, Billerica, MA, USA) and genetic analysis, i.e., sequence analysis of a PCR amplicon (using the ITS4 and ITS5 primers) of a ribosomal DNA locus (rDNA). A second swab, which was obtained from an abdominal drain as part of the routine screening on day 78, also yielded growth of this fungus, thereby disproving the hypothesis of an environmental contaminant.

Despite anti-infective therapy, several surgical and endoscopic interventions, and maximum escalation of intensive care treatment, the patient’s condition deteriorated. Regarding the poor prognosis, the therapy was changed for a palliative approach in consultation with the patient’s family, and the patient deceased on day 79. The relatives refused an autopsy.

**Fig. 1:**
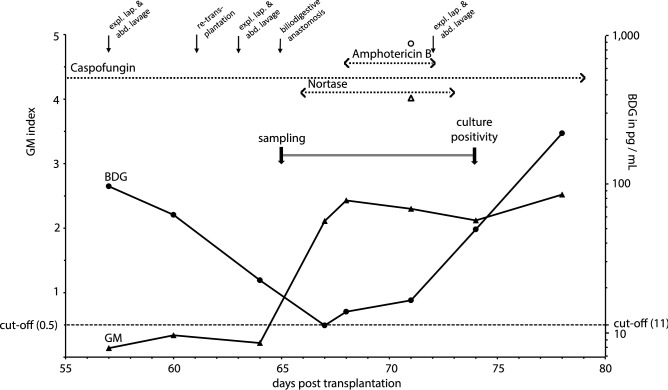
Time course of serology, antifungal therapy, and abdominal surgical procedures. Results of GM (full triangles, primary y-axis) and BDG (full circles, secondary y-axis) antigen testing from serum were plotted according to the time course of infection (x-axis: days post transplantation). Empty triangle and circle represent the respective test result of peritoneal fluid. Y-axes were scaled to allow to display a single cut-off line for both assays (dashed line). Dotted lines and the doubled line indicate the duration of drug administration and the time to positivity of *T. thermophilus* cultivation, respectively. expl. lap. & abd. lavage, exploratory laparotomy and abdominal lavage

Since the cause of antigenemia remained unclear, additional analyses were performed afterwards. GM positivity of Nortase® was confirmed by analysis of capsules of different batches. In addition, testing for the fungal cell wall polysaccharide β-1,3-d-glucan (BDG; Fujifilm Wako Chemicals Europe, Neuss, Germany) detected high amounts of BDG (up to 0.2 µg per capsule). The peritoneal fluid and all analyzed sera were BDG positive (Fig. 1). Notably, BDG antigenemia occurred already more than a week before Nortase® administration (Fig. 1).

**Fig. 2:**
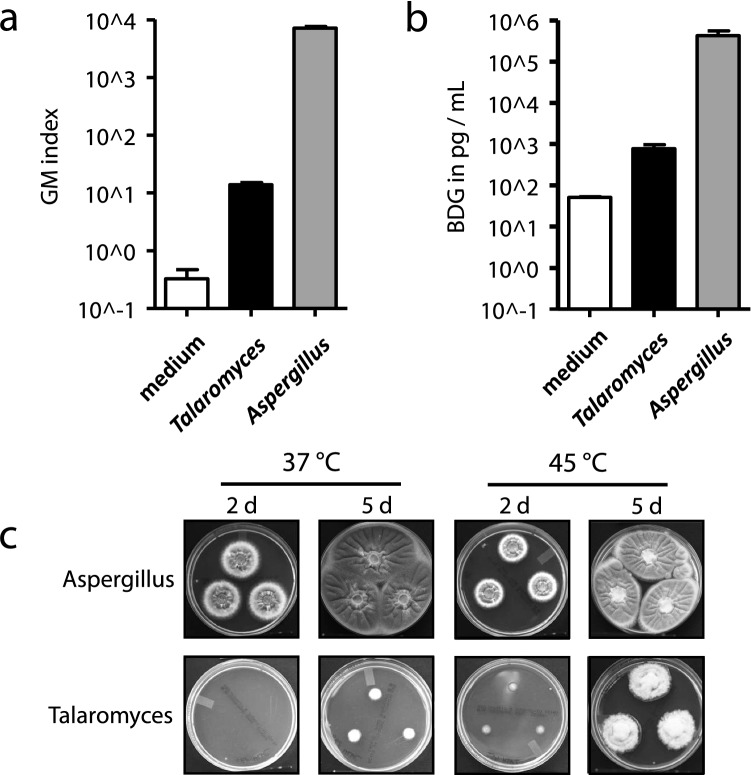
Comparison of in vitro antigen production and growth of *A. fumigatus* and *T. thermophilus*. Culture supernatants of *A. fumigatus* (strain D141) and the isolated *T. thermophilus* strain were analyzed for GM (**a**) and BDG (**b**). 500 conidia of the respective strains were inoculated on Sabouraud medium and incubated under the indicated conditions (**c**)

To address the question, whether the GM ELISA or the BDG assay is capable of detecting *T. thermophilus* infection, in vitro antigen production of the isolated strain was assessed. Briefly, 5*10^6 conidia / mL of *T. thermophilus* and of an *Aspergillus fumigatus* control (patient isolate D141) were incubated in 10 mL fetal bovine serum for 72 h at 37 °C. The culture supernatants were centrifuged, sterile filtered and subsequently tested for BDG and GM analogously to serum samples. If necessary, supernatants were diluted in medium in order to allow quantification of the results. In comparison to the medium control, *T. thermophilus* supernatants yielded a 42-fold reactivity in the GM ELISA and a 15-fold higher concentration of BDG (Fig. 2a, b)*.* As expected, results in both assays were by far higher measuring the *Aspergillus* supernatants (514-fold for GM and 544-fold). It is highly likely that much of this significant difference can be attributed to the massively impaired and delayed growth of *T.* *thermophilus* at 37 °C (Fig. 2c: 500 conidia of the respective strains were inoculated on Sabouraud medium [Sabouraud G + C, Becton Dickinson, Franklin Lakes, NJ, USA]). Notably, the thermophilic fungus failed to grow at 30 °C at all (data not shown).

Antifungal susceptibility testing of *T. thermophilus* was performed according to EUCAST recommendations for *Aspergillus* spp. with minor modifications taking into account the specific characteristics of the thermophilic organism (45 °C for 48 h; Sensititre YeastOne YO10 assay [TREK Diagnostic Systems, Cleveland, OH, USA]) [11]. The minimal inhibitory / effective concentrations (MIC / MEC) are listed in Table 1. Applying the *A. fumigatus* cut-offs, the *T. thermophilus* isolate was susceptible to amphotericin B, posaconazole, and voriconazole. The echinocandin MECs, for which no *Aspergillus* epidemiological cut-offs (ECOFFs) are available, were low and suggestive of susceptibility (0.015 µg / mL).

**Table 1:** Results of antisusceptibility testing

Antifungal agent	MIC (mg / l)	Interpretation
Fluconazole	> 256,000	R
Posaconazole	0.030	S
Voriconazole	0.015	S
Amphotericin B	0.250	S
	MEC	
Anidulafungin	0.015	–
Caspofungin	0.015	–
Micafungin	0.015	–

*MIC* minimal inhibitory concentration, *MEC* minimal effective concentration

## Discussion

Over the last decades, the incidence of IMI has risen, which can primarily be attributed to advances in immunosuppressive therapies and, thus, a growing risk population [1, 3–5, 7]. Notably, an emergence of infections with rare filamentous fungi was observed over the recent years [2–7, 12–15]. Due to climate change and global warming, appearance of new fungal pathogens or spread of endemic mycoses to new areas is expected [16, 17]. An infamous example for an (so far) endemic pathogen is the mold still known today as *Penicillium marneffei*, which is a common cause of severe and often fatal infections in AIDS patients in Southeast Asia [18, 19]. Notably, *P. marneffei* was reclassified in the genus *Talaromyces* in 2011 (*T. marneffei*) [20]. To date, virtually all infections caused by *Talaromyces* can be attributed to this single species, and, consequently, the term “talaromycosis” is used exclusively for fungal disease due to *T. marneffei* [18, 19].

We report to our knowledge the first case of an infection due to another member of the genus *Talaromyces*: the thermophilic organism *T. thermophilus*. It is a certain limitation of this case that no histopathology of the infected body site was available, which would represent the adequate diagnostic to exclude contamination with airborne spores or fungal cells originating from the gut. Due to the long incubation time after sampling, the finding of a mold was only available at a time point, when further invasive diagnostic procedures were dispensed with respect to the family’s consideration to change the therapy for a palliative treatment. However, the depicted case of a postoperative wound infection and peritonitis still meets the criteria of proven infection according to the EORTC/MSG consensus definitions of invasive fungal disease [21].

For the longest time, *T. thermophilus* has been regarded as an environmental saprophyte, which degrades organic materials in warm and humid environments, e.g., piles of agricultural and forestry products, tolerating temperatures of 50–60 °C [22]. Scientific interest in this mold has been limited to the biotechnological setting because of its thermostable enzymes [22, 23]. The definitive taxonomic status of *T. thermophilus* is still subject of outstanding investigations [24]. Based on phenotypic and genetic findings, a reclassification from the genus *Talaromyces* to the genus *Thermomyces*, which does not comprise any human pathogenic species at all, was proposed.

A major cause, if not the main cause, of the low pathogenicity of *Thermomyces* spp. is their inability to grow at 37 °C, since these organisms prefer higher temperatures. It is generally accepted to apply the term thermophilic, if organisms tolerate high temperatures or if high temperatures even provide the optimal growth condition. However, the exact limits defining “thermophile” are controversial and remain to be determined. Infections with thermophilic fungi are extremely rare with only few anecdotal reports published, e.g., mycoses due to *Thermoascus crustaceus* and *Myceliophthora thermophila* [25–30]. However, these fungi are cultivated reliably under standard laboratory conditions (30–37 °C). In contrast, in vitro growth of *T. thermophilus* is impeded at 30 °C and significantly impaired and delayed at 37 °C (data not shown and Fig. 2c), which makes mycological diagnostics challenging. It therefore seems conceivable that mycoses caused by thermophiles could be underreported due to these limitations. Furthermore, even upon isolation, the finding of *T. thermophilus* might be misinterpreted as contamination.

In contrast to culture, testing for fungal antigens might be a valuable diagnostic tool. The initial suspicion of IMI was based on a positive GM serology. The GM ELISA is an *Aspergillus*-specific test known for its high specificity and almost negligible cross reactivity to other fungal pathogens [31]. However, the close phylogenetic relationship of the genera *Talaromyces* and *Aspergillus* might be an explanation for this finding [24]. Testing for GM in culture supernatants demonstrated that *T. thermophilus* is capable of producing GM (or at least cross-reactive antigens) in vitro (Fig. 2a). The massively impaired growth at 37 °C could explain the lower amount of GM and BDG detected in the supernatant of the *T. thermophilus* isolate. Retrospective analysis of the panfungal antigen BDG in the patient’s sera yielded positive results throughout the entire course of infection and, notably, occurred ten days before GM seropositivity: from the time point of BDG antigenemia, three additional sera had to be sampled until the GM ELISA turned positive (Fig. 1). The finding of a temporal advantage of BDG over GM has been repeatedly observed [32, 33]. However, BDG testing does not allow a discrimination between a range of different fungal infections. Our patient had a history of intestinal colonization with *Candida*; therefore, it cannot be excluded that, as a result of mucosal leakage, he suffered from a parallel intra-abdominal *Candida* infection causing BDG antigenemia. BDG and GM were also detected in peritoneal fluid (Fig. 1). Anecdotal reports and small case series previously suggested that this analysis might be helpful in the setting of intra-abdominal fungal infections [34–36].

Interpretation of serological results was further complicated by the finding that the patient received at least one drug contaminated with both fungal antigens (Fig. 1): due to the pancreatectomy, digestion enzymes had to be substituted. The administered capsulated drug, i.e., Nortase® contains a mixture of lipase, amylase, and protease produced by *Rhizopus oryzae* and *A. oryzae*. Very recently, a dramatic increase of false-positive GM-results in patients receiving Nortase® was reported with 75% of treated individuals testing positive for GM [10]. A whole range of fungi is employed in the industrial production of intravenous drugs, so that GM contamination has been observed repeatedly: examples also include the β-lactam antibiotics piperacillin and meropenem as well as the antifungal agent caspofungin [37, 38]. All three substances had been administered to the patient as part of the treatment of his infectious complications.

Results of our antifungal susceptibility testing (Table 1) suggested that the isolated *T. thermophilus* strain was susceptible to echinocandins, voriconazole, and amphotericin B. However, no breakpoints have been defined for *Talaromyces* species, and testing of thermophilic species like in this case cannot be performed applying standard conditions as suggested by the EUCAST [11]. Furthermore, it is not clear whether the in vitro results reflect the in vivo situation. It is important to note that the *Talaromyces* IMI occurred under caspofungin treatment.

The current and the previously reported cases of thermophile infections demonstrate that clinicians must be aware of infections due to rare fungal pathogens and that such infections are not limited to hemato-oncological patients [25–30]. Furthermore, laboratories must consider that standard culturing conditions and procedures may fail to identify rare and emerging pathogens with special growth requirements, e.g., prolonged incubation or temperatures other than 37 °C. Antigen testing might be a helpful tool to compensate the limitations of culture-based diagnostics.

## Data Availability

Not applicable.
